# Effect of Long-Term Consumption of Poultry Egg Products on Growth, Body Composition, and Liver Gene Expression in Zebrafish, *Danio rerio*

**DOI:** 10.1093/cdn/nzab134

**Published:** 2021-12-24

**Authors:** Michael B Williams, Joseph W Palmer, Sophie B Chehade, Alex J Hall, Robert J Barry, Mickie L Powell, Melissa L Harris, Liou Y Sun, Stephen A Watts

**Affiliations:** Department of Biology, University of Alabama at Birmingham, Birmingham, AL, USA; Department of Biology, University of Alabama at Birmingham, Birmingham, AL, USA; Department of Biology, University of Alabama at Birmingham, Birmingham, AL, USA; Department of Biology, University of Alabama at Birmingham, Birmingham, AL, USA; Department of Biology, University of Alabama at Birmingham, Birmingham, AL, USA; Nutrition Obesity Research Center, University of Alabama at Birmingham, Birmingham, AL, USA; Department of Biology, University of Alabama at Birmingham, Birmingham, AL, USA; Nutrition Obesity Research Center, University of Alabama at Birmingham, Birmingham, AL, USA; Department of Biology, University of Alabama at Birmingham, Birmingham, AL, USA; Department of Biology, University of Alabama at Birmingham, Birmingham, AL, USA; Nutrition Obesity Research Center, University of Alabama at Birmingham, Birmingham, AL, USA; Department of Biology, University of Alabama at Birmingham, Birmingham, AL, USA; Nutrition Obesity Research Center, University of Alabama at Birmingham, Birmingham, AL, USA

**Keywords:** zebrafish, egg, nutrition, diet, transcriptome, blood glucose

## Abstract

**Background:**

Poultry eggs are a low-cost, high-protein nutrient package that can be consumed as part of quality diets. However, consumption of poultry egg products is historically contentious, which highlights the importance of investigating impacts of long-term egg consumption on metabolic health.

**Objective:**

Our study utilized the zebrafish, *Danio rerio*, a newly defined model of human metabolic health, to understand the metabolic consequence of consuming egg products in lieu of other well-described protein sources.

**Methods:**

Reference diets were formulated to contain multisource protein with casein and fish protein hydrolysate (CON; control protein sources), the protein sources that have been historically utilized in numerous reference diets. These proteins were then partially replaced with either whole egg (WE; protein and lipid source), egg white (EW; protein source), wheat gluten (WG; cereal protein source), or a high-lipid-content diet containing a multisource protein with casein and fish protein hydrolysate (HFCON; isonitrogenous and isolipidic with the WE diet) in a 34-wk trial (*n *= 8 tanks, 10 fish per tank). Daily feeding was initiated at the early juvenile life stage and terminated at the late reproductive adult stage.

**Results:**

The amino acid composition of control versus egg product diets did not vary substantially, although methionine and lysine were apparently limiting in fish fed WG. At termination, fish fed EW as the protein source had weight gain and body composition similar to those fed the CON diet. Fasting and postprandial blood glucose did not differ between any dietary treatment. Assessment of the liver transcriptome using RNAseq revealed no differential gene expression between zebrafish fed CON or WE diets. Zebrafish fed WG had lower weight gain in males.

**Conclusions:**

Long-term consumption of egg products promoted metabolic health equal to that of historically relevant proteins. These data support the value of egg products for maintaining long-term metabolic health in animal diets.

## Teaser

Replacement of standard dietary protein sources with egg products results in no differences in blood glucose, growth, adiposity, or liver gene expression in zebrafish.

## Introduction

Protein is identified as one of the most important macronutrients in regulating growth and tissue production. In addition to promoting growth of lean tissue, protein quantity, quality, and source impact known markers of metabolic health. In humans, a modest increase in protein intake resulted in improved weight maintenance and glycemic index following a weight-loss regime (1). Eight weeks with an increased protein diet reduced systolic and diastolic blood pressure (2). Diets substituting protein for carbohydrate in individuals with insulin-resistant diabetes reduced blood glucose and circulating insulin in both males and females (3, 4). There is some evidence of an overall increase in risk of development of type 2 diabetes that is dependent on protein source (5). Increased animal protein intake, substituted for dietary carbohydrate, resulted in greater overall lifetime risk of type 2 diabetes development, but a modest reduction in this risk was seen when plant protein sources were supplemented instead. These observations suggest an impact of protein on human health from protein as a dietary source; however, the lack of consistency and standardization of diets among these studies is confounding. Branched-chain amino acid (BCAA) content, which is high in eggs and dairy protein sources, is also implicated in conferring reduced insulin resistance (6, 7). Circulating BCAA concentrations are associated with reduced insulin resistance and are proportional to dietary intake, although studies show a significant increase in blood glucose as well. Circulating concentrations of the protein zonulin, which reduces inflammation-related intestinal permeability (8), were inversely proportional to dietary protein intake. These and related studies confirm the importance of protein source and content as likely effectors of metabolic health and disease onset and progression.

In many populations, poultry eggs provide a valuable protein source containing essential macronutrients and other bioactive compounds known to promote health while supporting growth and development (9). Egg-derived bioactive compounds also confer antibacterial activity (10), antihypertensive activity (11), antioxidant capability, and mineral binding properties (12). Unfortunately, few studies have addressed the effects of long-term consumption of egg products as regulators of metabolic health in vertebrates, particularly across a series of life stages.

Direct testing of egg products by nutritional interventions is difficult and expensive to administer in human clinical trials. Ethical and economic concerns coupled with diverse and limited test populations reduce the opportunities to determine long-term nutritional management strategies for human health maintenance. For these reasons, we believe a rapid, low-cost, high-throughput evaluation model, the zebrafish, *Danio rerio*, can be used to screen various nutritional therapies as positive mediators of metabolic health (13). In the last decade, zebrafish have been identified as a relevant model for the study of human nutrition, as they are an omnivore with similar protein and lipid requirements (14). Zebrafish exhibit diet-induced obesity [DIO (15)] and have similar genomic responses as seen in human DIO (16). Additionally, zebrafish show obesity-related comorbidities such as type 2 diabetes (17). Importantly, we are able to formulate zebrafish diets that contain egg products and compare age- and size-related development as young juvenile zebrafish cohorts transition to sub-adult and reproductive adult populations. In this study we compare a typical zebrafish diet containing fish and casein protein with diets containing egg products. A diet containing a cereal-based protein source was provided for comparison.

## Methods

### Husbandry

Zebrafish were bred, housed, and treated in compliance with the Institute for Laboratory Animal Research Guide for the Care and Use of Laboratory Animals, and the protocol has been approved by the University of Alabama at Birmingham Institutional Animal Care and Use Committee (IACUC-20656). Adult fish were held at the University of Alabama at Birmingham Nutrition Obesity Research Center Lab Animal Nutrition Core in a recirculating zebrafish husbandry system (Aquaneering) at 28°C and 14 h:10 h light:dark cycle. Municipal tap water was filtered by a 5-μm string filter, followed by charcoal and reverse osmosis, and passed through an ion-exchange column to ensure the highest quality of system water. Ions are provided by the addition of synthetic sea salt (Instant Ocean) to a salinity of 0.7 ppt (∼1500 μS/cm). Water quality was monitored twice weekly (ammonia, nitrite, nitrate) and pH daily (7.4, adjusted with sodium bicarbonate). Embryos of multi-individual crosses (10 males and 20 females) were held at standard conditions (Zebrafish International Resource Center, Eugene, OR, USA). At 5 [days post-fertilization (dpf)], populations with inflated swim bladders were placed in 5-ppt salinity system water and fed in polyculture with the rotifer *Brachionus plicatilus* ad libitum (*B. plicatilus* were reared on high unsaturated fatty acid-fortified *Nannochloropsis*; Reed Mariculture). At 10 dpf, individuals (larvae) were fed stage I nauplii of *Artemia* (INVE Aquaculture) and assigned randomly to experimental tanks (2.8-L tanks, 10 fish per tank, 8 replicate tanks per diet treatment). This density promotes excellent growth characteristics and assures an even sex ratio [Watts SA (2013). Population density affects zebra fish growth and sex ratios, unpublished manuscript]. At 21 dpf, young juvenile cohorts were fed (twice daily at 08:00 and 16:00) experimental diets (see below). All formulated diets were fed at ∼ 6% body weight per day, a ration amount shown to promote weight gain. Experimental tanks were provided their respective diet for the study period. At the end of 34 wk of being fed these diets, reproductively active adult zebrafish were terminated by approved rapid ice immersion. Mixed-sex fish (*n *= 10) from random tanks of each dietary treatment were returned to the recircuiting system for blood glucose testing (see below). Sexes were easily identified at termination, and fish of each sex were randomly selected for lipid analysis and gene expression of the liver, as described below. Power analyses for experimental design and post hoc statistics were provided by the University of Alabama at Birmingham Nutrition Obesity Research Center Statistical Core personnel. The treatment period encompassed zebrafish beginning as early juveniles until several months after reproductive maturity (older adults).

### Diet preparation

All ingredients were weighed on an analytical balance (Mettler Toledo New Classic MF Model MS8001S or Model PG503-S; Mettler-Toledo, LLC) and mixed using a Kitchen Aid Professional 600 Orbital Mixer (Kitchen Aid); the ingredients and catalog numbers are listed in **Table 1**. The diets were cold extruded into strands to preserve nutrient content using a commercial food processor (Cuisinart), and strands were air-dried for 24 h on wire trays to a final moisture content of ∼8%. Protein sources were either fish protein hydrolysate (catalog no. CPSP90; The Scoular Company), casein (catalog no. 904,798; MP Biomedicals), isolated soy protein (catalog no. 905,456; MP Biomedicals), wheat gluten (WG; catalog no. G5004; Sigma Aldrich), egg white (EW; catalog no. 2110; Ballas Egg Products Corp.), or whole egg (WE; catalog no. 3010; Ballas Egg Products Corp.). Diets included *1*) a control diet [CON; a multisource protein diet shown previously to provide lifelong health and growth (15)]; *2*) a diet in which 50% of the total protein is substituted with WG, isonitrogenous to the control; *3*) a diet in which 50% of the total protein is substituted with EW, isonitrogenous to the control; *4*) a diet with WE substituted for ∼50% of the total protein; and *5*) an elevated-fat diet (HFCON) in which total protein and fat were isonitrogenous and isolipidic with the replacement of 50% of the total protein with the WE diet (**Table 2**). The amino acid compositions of these respective diets are shown in **Table 3**. The WG diet was provided as a comparative commercial, egg-free, cereal-based diet.

**TABLE 1:** Vendors and catalog numbers of ingredients

Ingredient	Vendor	Catalog identifier
Dried whole egg	Ballas Egg Product Corp.	3010
White egg	Ballas Egg Product Corp.	2110
Fish protein hydrolysate	The Scoular Company	CPSP90
Wheat gluten	MP Biomedicals	101815
Dextrin	Acros Organics	406285000
Mineral mix AIN 76	Envigo	CA.170915
Casein low-trace metals	MP Biomedicals	0296012805
Soy protein, isolated	MP Biomedicals	0290545605
Corn oil	MP Biomedicals	0290141401
Safflower oil	MP Biomedicals	0210288890
Menhaden fish oil	Omega Protein	Virginia Prime Gold
Vitamin diet fortification mixture	MP Biomedicals	0290465401
Diatomaceous earth, acid washed	Andwin Scientific	D3877
Alphacel, nonnutritive bulk	MP Biomedicals	0290045305
d-(+)-glucosamine hydrochloride	MP Biomedicals	0210178225
Cholesterol National Formulary grade	MP Biomedicals	02101380-CF
Lecithin, soy, refined	MP Biomedicals	0210214790
Ascorbyl palmitate	MP Biomedicals	0210078180
Potassium phosphate monobasic	MP Biomedicals	02195453.5
Wheat starch	MP Biomedicals	0290295225
Alginate	TIC Gums	TICA-Algin 400
Betaine	MP Biomedicals	150461
Canthaxanthin	DSM	Carophyll Red

**TABLE 2:** Ingredient composition of diets (g/kg)^1^

Ingredient	CON	EW	WG	HFCON	WE
Casein, low-trace metals	217.5	130.5	130.5	217.5	130.5
Fish protein hydrolysate	217.5	130.5	130.5	217.5	130.5
Soy protein isolate	60	36	36	60	36
Egg white	—	217.6	—	—	—
Wheat gluten	—	—	232	—	—
Whole egg	—	—	—	—	352.3
Wheat starch	120	120	120	60.5	60.5
Dextrin	50	50	50	50	50
Diatomaceous earth	94.8	65.6	53	17.8	—
Alginate	20	20	20	20	20
Safflower oil	40	46.4	45.2	131	40
Menhaden fish oil (ARBP)	20	23.2	22.6	65.5	20
Soy lecithin (refined)	40	40	40	40	40
Vitamin mix (MP-VDFM)^2^	40	40	40	40	40
Mineral mix (AIN 76)^3^	30	30	30	30	30
Canthaxanthin (10%)	10	10	10	10	10
Potassium phosphate monobasic	11.5	11.5	11.5	11.5	11.5
Alpha cellulose	23.1	23.1	23.1	23.1	23.1
Glucosamine	2.5	2.5	2.5	2.5	2.5
Betaine	1.5	1.5	1.5	1.5	1.5
Cholesterol	1.2	1.2	1.2	1.2	1.2
Ascorbyl palmitate	0.4	0.4	0.4	0.4	0.4
Calculated protein (% as fed)	41.97	40.87	41.38	41.95	39.45
Calculated protein (% dry)	46.64	45.41	45.98	46.62	43.83
Calculated lipid (% as fed)	10.12	10.43	10.48	22.40	22.24
Calculated lipid (% dry)	11.25	11.59	11.64	24.89	24.71
Calculated soluble digestible carbohydrate (% as fed)^4^	33.00	37.19	37.36	27.70	32.72
Ash (% as fed)	10.20	6.96	6.03	4.69	1.04
Fiber (% as fed)	4.70	4.56	4.75	3.26	4.55
Protein energy ratio (as fed)	0.558	0.527	0.529	0.456	0.425
Calculated energy (kcal/kg as fed)	4248	4382	4422	5195	5239

^1^All ingredients are listed by g/kg in the diet as fed. Dietary protein percentages are based on crude protein values of each protein source as provided by the manufacturer. ARBP, alkali refined bleached and pressed; CON, fish and casein protein source control diet; EW, egg white; HFCON, high-fat fish and casein protein source diet; MP-VDFM, MP Biomedicals™ Vitamin Diet Fortification Mixture; WE, whole egg; WG, wheat gluten.

^2^MP Biomedicals 904654: vitamin A acetate (500,000 IU/g) 1.80000, vitamin D_2_ (850,000 IU/g) 0.12500, dl-ɑ-tocopherol acetate 22.00000, ascorbic acid 45.00000, inositol 5.00000, choline chloride 75.00000, menadione 2.25000, p-aminobenzoic acid 5.00000, niacin 4.25000, riboflavin 1.00000, pyridoxine hydrochloride 1.00000, thiamin hydrochloride 1.00000, calcium pantothenate 3.00000, biotin 0.02000, folic acid 0.09000, vitamin B-12 0.00135; measures are mg/g.

^3^AIN 76 mineral mix for Envigo (Indianapolis, IN): sucrose, fine ground 118.03; calcium phosphate, dibasic 500.0; sodium chloride 74.0; potassium citrate, monohydrate 220.0; potassium sulfate 52.0; magnesium oxide 24.0; manganous carbonate 3.5; ferric citrate 6.0; zinc carbonate 1.6; cupric carbonate 0.3; potassium iodate 0.01; sodium selenite, pentahydrate 0.01; chromium potassium sulfate, dodecahydrate 0.55; measures are mg/g.

^4^Calculation used for soluble digestible carbohydrate: carbohydrate = 100 − (protein % + fat % + ash % + fiber %).

**TABLE 3:** Amino acid composition of diets^1^

Amino acids (g/100 g dry)	CON	EW	WG	HFCON	WE
Alanine	1.88	2.29	1.63	1.88	2.08
Arginine	2.52	2.56	2.18	2.52	2.60
Aspartic acid	3.10	3.45	2.49	3.10	3.63
Cystine	1.17	1.14	1.16	1.17	1.08
Glutamic acid	7.04	6.73	11.43	7.04	6.48
Glycine	2.26	2.02	2.01	2.26	1.93
Histidine	1.31	1.20	1.21	1.31	1.21
Isoleucine	1.90	2.23	1.83	1.90	2.00
Leucine	3.69	3.77	3.52	3.69	3.67
Lysine	3.31	3.09	2.30	3.31	3.16
Methionine	1.19	1.41	0.99	1.19	1.24
Phenylalanine	2.03	2.34	2.22	2.03	2.11
Proline	3.16	2.57	4.60	3.16	2.54
Serine	2.30	2.70	2.35	2.30	2.71
Threonine	1.72	1.83	1.58	1.72	1.78
Tryptophan	0.54	0.60	0.56	0.54	0.60
Tyrosine	3.00	2.52	2.43	3.00	2.50
Valine	2.54	2.86	2.27	2.54	2.57

^1^The amino acid composition was calculated based on amino acid composition of protein sources. The amino acid contents of soy isolate, fish protein hydrolysate, and casein protein sources were analyzed by Midwest Laboratories (Omaha, NE, USA). The amino acid contents of dry whole egg and egg whites were obtained from USDA Database for Standard References and wheat gluten amino acid content was obtained from the National Animal Nutrition Program Database. CON, fish and casein protein source control diet; EW, egg white; HFCON, high-fat fish and casein protein source diet; WE, whole egg; WG, wheat gluten.

### Body metrics

Every 2 wk following assignment to diets, survival and total tank fish weights were measured to estimate population biomass and to adjust feed ration (fish were fed at ∼6% of body weight per day). At 34 wk, the growth trial was terminated, and all fish were sexed, weighed individually to 0.001 g, and terminal standard body length (measured from tip of snout to the distal end of the caudal peduncle when laid sidewise on a ruler) was recorded to 0.1 mm. Females were ovariectomized and total lipid for the remaining female carcass and intact males (*n *= 10 per diet) was determined using the Folch lipid extraction optimized for zebrafish (18). Total body moisture was determined by oven-drying carcasses (72 h at 50°C) used for Folch analysis.

### Blood glucose

Mixed-sex fish (*n *= 10) from random tanks of each dietary treatment continued to be fed within their respective treatment protocols for an additional 4 wk (38 wk total). The fish were then monitored for fasting blood glucose as described previously (19). For fasting blood glucose, fish were feed-deprived for a 16-h period and anesthetized using ice water. For postprandial glucose measures, fish were fed a regular ration of assigned diet and allowed to consume the diet for a 30-min period. Blood was collected in heparinized glass pipettes punctured into the region of the dorsal aorta. Glucose concentrations in the samples were determined immediately following blood collections using Amplex Red Glucose Assay (Life Technologies).

### RNA isolation

At termination of the feeding trial, males and females (*n *= 6 of each sex) from the CON, WG, HFCON, and WE dietary treatments had whole livers dissected out, frozen in liquid nitrogen, and transferred to a –80°C freezer for long-term storage. RNA was isolated from tissue using RNeasy™ Lipid Tissue Mini Kit (Qiagen) per the manufacturer's instructions. Purified RNA was subjected to quantification and purity assessment via an Epoch microplate spectrophotometer (BioTek Instruments).

### RNA sequencing

Four female samples from the CON and WE dietary treatments were processed for RNA sequencing (RNAseq; Genomics Core Laboratory, Heflin Center for Genomic Sciences, University of Alabama at Birmingham). In brief, quality of the total RNA was assessed using the Agilent 2100 Bioanalyzer. RNA with an RNA Integrity Number (RIN) of 7.0 or above was used for sequencing library preparation using the Agilent SureSelect Strand Specific mRNA library kit as per the manufacturer's instructions (Agilent). Library construction began with ribosome reduction using the NEBNext rRNA depletion kit for human/mouse/rat as described by the manufacturer (New England Biolabs). The resulting RNA was randomly fragmented with cations and heat, which was followed by first-strand synthesis using random primers with inclusion of actinomycin D (2.4 ng/µL final concentration). Second-strand cDNA production was done with standard techniques; the ends of the resulting cDNA were made blunt, A-tailed, and adaptors ligated for amplification and indexing to allow for multiplexing during sequencing. The cDNA libraries were quantitated using RT-PCR in a Roche LightCycler 480 with the Kapa Biosystems kit for Illumina library quantitation (Kapa Biosystems) prior to cluster generation. Cluster generation was performed according to the manufacturer's recommendations for onboard clustering (Illumina). Samples were sequenced to achieve a minimum of 30 million, single-end, 75-bp reads per sample.

Quality of the sequencing reads was assessed using FastQC (version 0.11.8) (20) and the data were filtered for quality and rRNA contamination using Trimmomatic (version 0.36) (21). Sequences were aligned to the Ensembl zebrafish reference genome (GRCz11) using the full annotated (49 K genes) gtf file. Individual gene counts were obtained for each sample using the quantMode feature of the STAR aligner (version 2.5.2b) (22), with an average 76.5% of the total reads per sample uniquely mapping. The final read depth across all samples was ∼20–23 million reads per sample and the gene counts from these reads were used for differential gene expression analysis using DESeq2 (version 1.22.2) (23) and methods similar to those published previously by Harris et al. (24). For validation purposes, independent RNAseq analysis was performed by Sunil K Singh at the University of Iowa and used methods similar to those listed above, with the exception that the featureCounts (25) tool was used to count mapped reads per transcript. Principal component analysis (PCA) and Pearson correlation plots were made using the pcaExplorer tool (26).

### Real-time PCR

For real-time PCR validation, cDNA was synthesized using a High-Capacity cDNA Reverse Transcription Kit (Applied Biosystems) as per the manufacturer's instructions, with a starting amount of 5 μg total RNA in a 100-μL reaction run on a SimpliAmp™ Thermal Cycler (Applied Biosystems). The cDNA was diluted to 1:20 and 5 μL was used for a 20-μL total reaction using TaqMan™ Fast Advanced Master Mix (Applied Biosystems) and MicroAmp™ Fast Optical 96-Well Reaction Plates (Applied Biosystems). Gene-specific TaqMan primers were purchased from ThermoFisher (assay IDs: rpl13a-Dr03101114_gl, ucp2-Dr03125005_m1, elovl5-Dr03094287_m1, and gpx4a-Proprietary). Forty RT-PCR cycles were run on a QuantStudio 3 Real-Time PCR System and results analyzed with QuantStudio™ Design & Analysis Software version 1.5.1 using ribosomal protein L13a (*rpl13a*) as the housekeeping gene (Applied Biosystems).

### Statistical modeling and analysis

Data from this study were analyzed with RStudio Statistical Software (R Core Team, 2016, v0.99.896), and graphs generated with Statistical Package for Social Science (SPSS) version 26 (IBM Corporation). All analyses for continuous outcomes were stratified by sex, with the exception of glucose. Weekly and terminal body weights were analyzed by a linear mixed-effects model with tank as a random effect. Total body length, glucose measures, and liver gene expression were compared separately by ANOVA. Fat mass was analyzed with ANCOVA, adjusting for body weight as a covariate. Any observed significant differences (*P* < 0.05) were further analyzed with pairwise comparisons among diets using Tukey's honestly significantly different (HSD) post hoc test. All data were analyzed for normality and equal variances. Any datasets with a nonnormal distribution were log-transformed. RNAseq data are available at NCBI GEO, accession #GSE184565.

## Results

**FIGURE 1:**
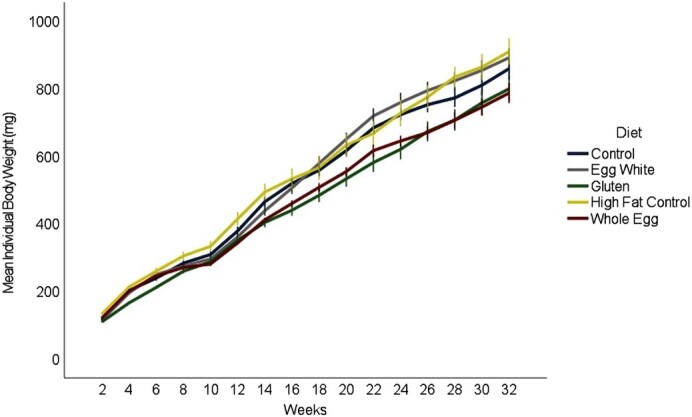
Mean ± SE (indicated by bars) of body weight for individual fish (mg) for male and female zebrafish (combined) measured every 2 wk on the assigned diets (*n *= 8 tanks, 10 fish per tank for each diet treatment).

**FIGURE 2:**
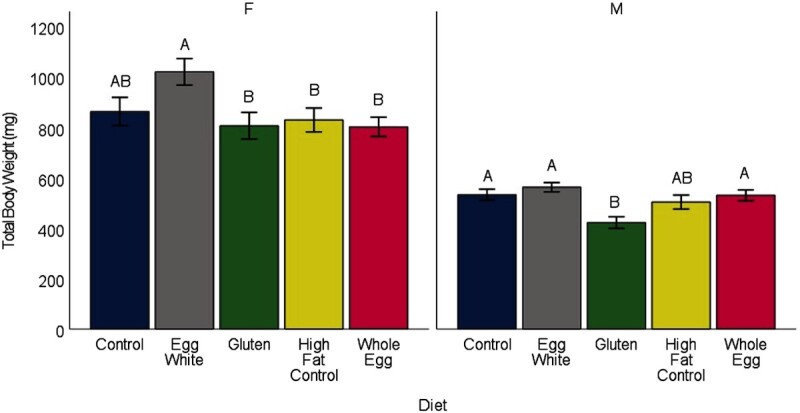
Mean ± SE (indicated by bars) of total body weight (mg) for male (M) and female (F) zebrafish measured at the end of 34 wk on the assigned diets (*n *= 8 tanks, 10 fish per tank for each diet treatment). Within each sex, different uppercase letters indicate differences between dietary treatments (*P* < 0.05).

**FIGURE 3:**
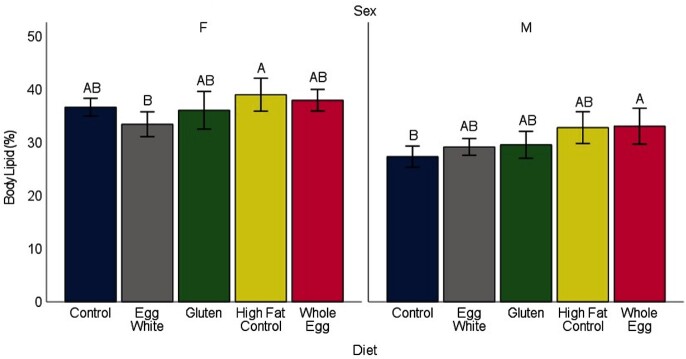
Mean ± SE (indicated by bars) of total body lipid (%) for male (M) and female (F) zebrafish measured at the end of 34 wk on the assigned diets (*n *= 10 males and 10 females from random tanks for each diet treatment). For each sex, different uppercase letters indicate differences between milligrams of lipid for each dietary treatment at *P* < 0.05 covaried by dry body weight.

**FIGURE 4:**
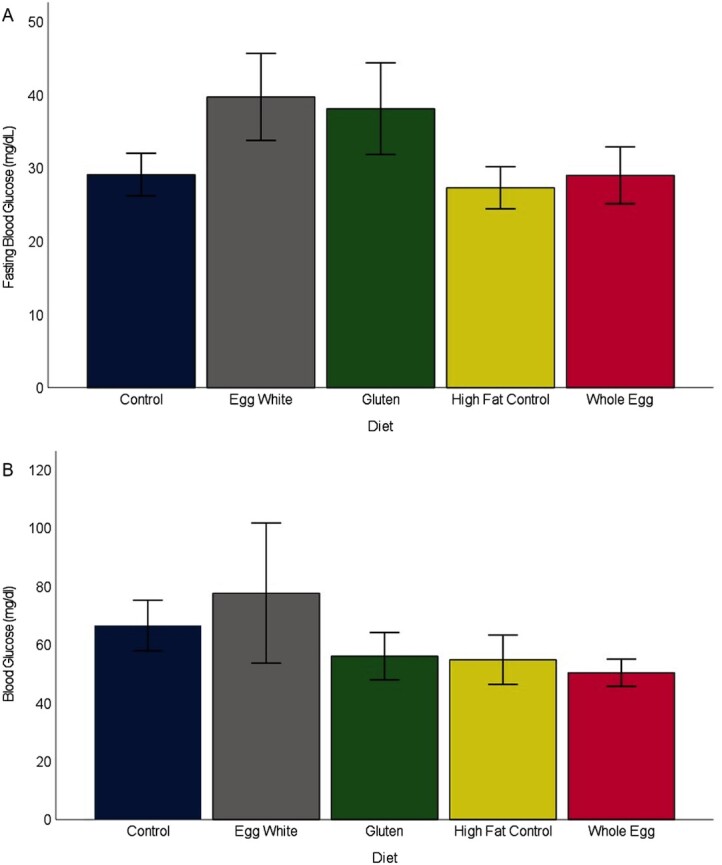
Mean ± SE (indicated by bars) of fasting (A) and postprandial (B) blood glucose measures (mg/dL) for male and female zebrafish (combined) measured at the end of 38 weeks on the assigned diets (*n *= 10 mixed sex males and females from random tanks for each diet treatment). There were no statistically significant differences between treatments.

Fish in all treatments showed patterns of weight gain and body metrics that were indicative of fish with a normative phenotype. Although reproductive success was not tested in this study, female fish from all diet treatments had a body shape typical of normal reproductive females at the same age as those at study termination, and dissection revealed mature ovaries. The biweekly weights showed differences between the diets consisting of WG and EW (*P* = 0.009), WG and HFCON (*P* < 0.001), and WE and HFCON (*P* = 0.014) (**Figure 1**). Male terminal weights showed WG-fed fish weighed less than the EW-, WE-, and CON-fed fish (*P* < 0.009) (**Figure 2**). Female terminal weights showed EW-fed fish weighed more than WE-, HFCON-, and WG-fed fish (*P* < 0.045). Terminal body lengths showed no differences between any diet group for males or females (*P* > 0.385) (**Supplemental Figure 1**). Body moisture showed no differences between any diet group for males or females (*P* > 0.421) (**Supplemental Figure 2**). Total body lipids showed a difference in females, with fish fed HFCON having a higher total body lipid concentration than those fed EW (*P* = 0.027), and in males, with fish fed WE having a higher total body lipid concentration than those fed CON (*P* = 0.043) (**Figure 3**). No differences were observed in feed-deprived (*P* > 0.273) or postprandial (*P* > 0.663) blood glucose between any of the diet treatments with males and females analyzed together (**Figure 4**).

**FIGURE 5:**
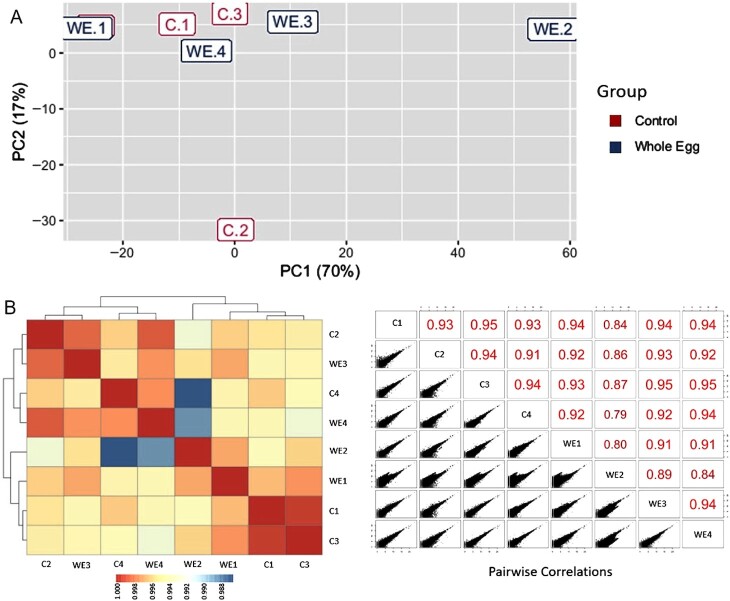
Principal component analysis of the gene expression from RNAseq Control (C.1–C.4) and Whole Egg (WE.1–WE.4) samples, including all samples (A). Pearson correlation analysis for correlation between Control and Whole Egg samples based on RNAseq data, including all samples (B). PC, principal component; RNAseq, RNA sequencing.

**FIGURE 6:**
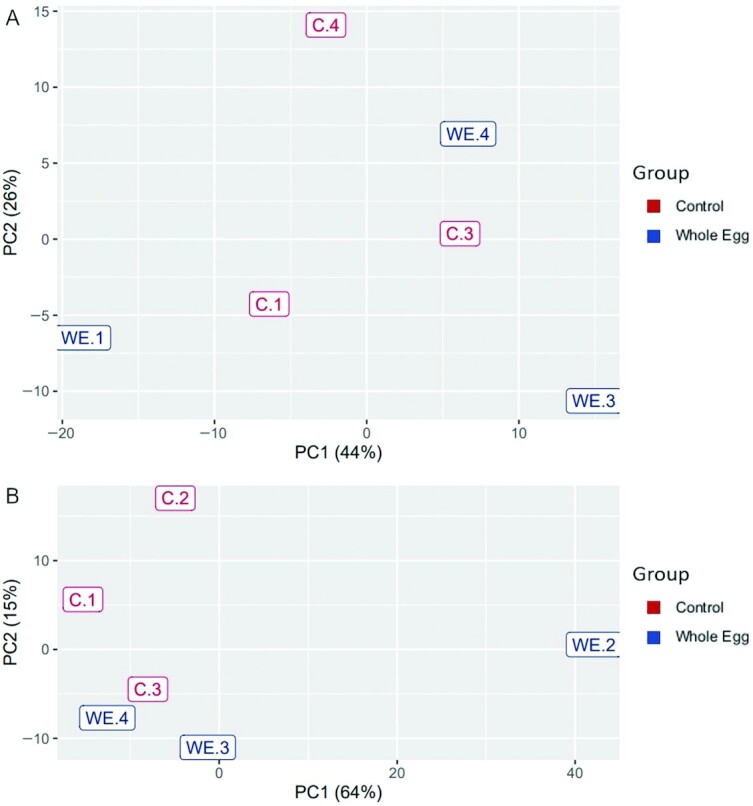
Principal component analysis of the gene expression from RNAseq Control (C.1–C.4) and Whole Egg (WE.1–WE.4) samples excluding outlier samples C.2 and WE.2 (A) or the overlapping samples C.4 and WE.1 (B). PC, principal component; RNAseq, RNA sequencing.

**FIGURE 7:**
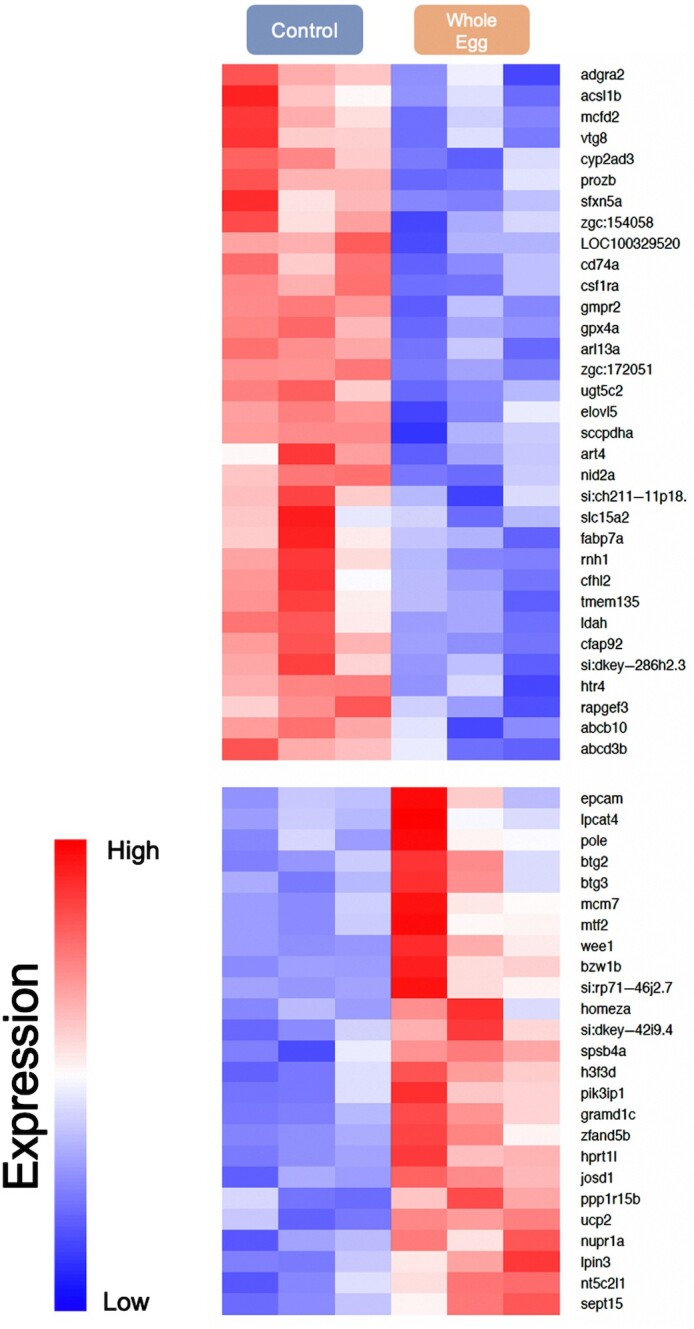
Heatmap of expression of 62 DEGs from Control and Whole Egg samples after RNAseq analysis, excluding samples Control-4 (C.4) and Whole Egg-1 (WE.1), which overlapped. DEG, differentially expressed gene; RNAseq, RNA sequencing

RNAseq analysis was used to evaluate global changes in liver transcriptomics in response to long-term feeding on the CON and WE diets. PCA of the regularized log-transformed count values (rlog) for liver gene expression of CON and WE dietary treatments across samples revealed that the majority of the samples clustered together, irrespective of diet (**Figure 5**A; other diet treatments were not analyzed). Additionally, no differentially expressed genes (DEGs) could be detected when comparing gene expression between diets (adjusted *P* < 0.1). Similar results were obtained after independent RNAseq analysis (performed by Sunil K. Singh at the University of Iowa). Pearson's correlation analysis using rlog-transformed data revealed that the correlation between samples of different diets was very similar to the correlation between samples of the same diet (Figure 5B). While these observations suggest that the experimental dietary changes compared here do not elicit detectable changes in gene expression within whole liver tissue, PCA also highlighted 2 outliers from each diet that contributed to the majority of the observed variance in the PCA plot (Figure 5A; CON-2 and WE-2). Whether biological or technical outliers, removal of CON-2 and WE-2 did not improve the PCA clustering (**Figure 6**A), and differential expression analysis of the remaining samples again revealed no significant DEGs (adjusted *P* < 0.1). Alternatively, in the interest of identifying the DEGs responsible for the majority of variance represented by the samples, 2 overlapping samples from the different diets (CON-4 and WE-1) were removed. Genes with read counts less than 30 were also excluded to increase sensitivity. PCA of this adjusted dataset revealed modest separation of samples by diet, particularly across PC2 (Figure 6B). When comparing these remaining samples, differential gene expression analysis identified 62 DEGs between fish fed the WE and CON dietary treatments (log-2 fold-change >0.8; adjusted *P* < 0.1; **Supplemental Data File**). Of these DEGs, 36 were downregulated and 26 upregulated in the WE diet compared with the CON diet (**Figure 7**). Three DEGs were further validated in a larger sample set—uncoupling protein 2 (*ucp2*), glutathione peroxidase 4a (*gpx4a*), and elongation of very long chain fatty acids protein 5 (*elovl5*) (log-2 fold-change = 1.25, –1.04, and –1.11, respectively).

**FIGURE 8:**
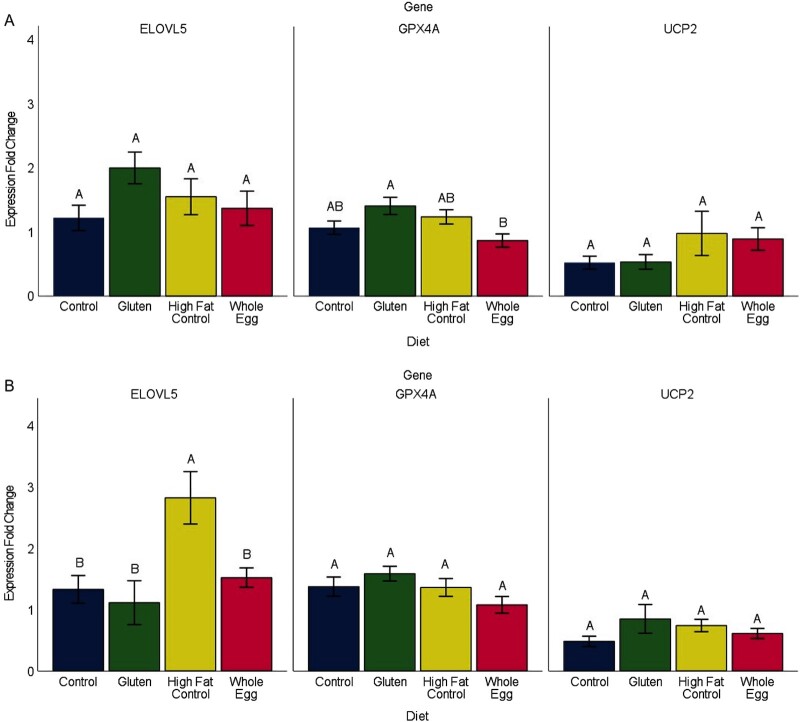
Mean female (A) and male (B) mean liver gene expression ± SE (indicated by bars), normalized to ribosomal protein L13a (*rpl13a*) expression, measured at the end of 34 wk on the assigned diets (*n* = 4–6 livers). Different uppercase letters indicate differences between dietary treatments (*P* < 0.05). ELOVL5, elongation of very long chain fatty acids protein 5; GPX4A, glutathione peroxidase 4a; UCP2, uncoupling protein 2.

Using RT-PCR and RNA from the same female samples evaluated by RNAseq, we found similar trends in gene expression between the WE and CON diets across all 3 genes (**Supplemental Figure 3**). However, expanding to additional female samples and across sexes (male) did not further corroborate the significance of the DEGs derived from our RNAseq dataset (**Figure 8**). No difference in liver gene expression was observed between females of the CON and WE dietary treatments for *gpx4a* (*P* = 0.461), *elovl5* (*P* = 0.972), or *ucp2* (*P* = 0.360) (Figure 8A). Similarly, liver gene expression in males of the CON and WE dietary treatment showed no difference for *gpx4* (*P* = 0.383), *elovl5* (*P* = 0.960), or *ucp2* (*P* = 0.877) (Figure 8B). Interestingly, across these 3 genes and additional diets, we did detect increased expression of *gpx4a* in females fed the WG diet compared with the WE diet (*P* = 0.0177) and increased expression of *elovl5* in males fed the HFCON diet compared with the CON, WG, and WE diets (*P* < 0.0262).

## Discussion

Overall, our data support the value of poultry egg consumption for maintaining long-term metabolic health in zebrafish, as assessed by weight gain, body composition, glycemic response, and liver transcriptomics. The consistent increase in body weight among all treatments shows that zebrafish consuming egg protein were able to sustain normal patterns of growth over this portion of the lifespan as compared with fish meal hydrolysate (fish meal is considered the gold standard of protein for typical fish diets and in NIH-31 rodent diets) and casein (a highly purified and well-defined protein source). The lack of significant body weight differences between diets containing egg products indicate egg product replacement of quality protein sources supports fish maturation and health. These data would suggest that amino acids provided by egg products are sufficient and not limiting when compared with fish protein and casein. In fact, total amino acid profiles were very similar in zebrafish fed fish protein, casein, and egg products. Male fish fed WG did not maintain the same body weight gain as male fish fed egg products or fish/casein protein, despite receiving the same amount of crude protein. An evaluation of the amino acid content of the WG diet shows methionine and lysine were lower than in all other diets. Since methionine and lysine can be rate limiting for growth, we hypothesize the cereal protein diet may be nutritionally limited for zebrafish, similar to what is observed in rodents. For both sexes, the EW diet fish were significantly larger than the fish fed the WG diet, with no differences in body lipid between these diets. This suggests that the EW diet is promoting more lean body mass growth than the WG diet, furthering the hypothesis that protein quality and amino acid content are important. Historically, WG-based diets can have detrimental effects on animal health, including humans (27).

The WE and HFCON diets were formulated to contain the same caloric fat content, both of which had a higher energy content than the lower-fat CON diet. Feed intake could not be measured appropriately and, thus, nutrient (or caloric) intake could not be estimated. We did not observe a difference in adiposity between the EW diet fed fish and any of the other diets. This supports the hypothesis that higher body lipids for WE-diet–fed males were derived from the increased fat (caloric) content of the diet, which is a consequence of using WE (with yolk) as a protein source.

Moisture content of a fish carcass is often predictive of body composition and physiological status. The lack of body moisture differences between diet treatments suggests similar water retention or osmotic regulation, an issue observed in some cases of metabolic syndrome and obesity (28). The water content observed here is also comparable to studies across teleost species (29).

Excluding protein, additional differences in the ingredient composition and, thus, nutrient content of the egg product diets versus the control diet could be considered substantial. Although total protein nitrogen and total lipid content were balanced and comparable, the nutrient quantity was different, including small changes in BCAAs, fatty acid profiles, cholesterol, and unnamed bioactive food components. Despite these differences in dietary nutrient content, both feed-deprived or postprandial blood glucose concentrations were similar in fish consuming all diets. Several of the diets also contained additional wheat starch to balance the macronutrient and energy content in the diet formulation. Diets of higher wheat starch (CON, WG, and EW) also show no differences in blood glucose metrics compared with those with less or no wheat starch (HFCON and WE). It is possible that egg products were preventative or therapeutic in maintaining glycemic metrics and, thus, metabolic homeostasis.

Due to resource limitations, we were only able to evaluate differences in liver gene expression between CON- and WE-fed fish by RNAseq. Differences in gene expression between these diets were not apparent, despite a dramatic difference in the ingredient source of proteins and fat. DEGs that were detected when the stringency of the RNAseq analysis was relaxed were not representative of the larger sample populations when evaluated by RT-PCR. From these observations we conclude that the substitution of WE into the diet of zebrafish does not produce adverse changes in liver transcriptomics in comparison to a diet with fish meal and casein as the main protein sources. Fish meal and casein proteins are considered the highest in quality in most fish diets (30), and these data support the fact that long-term consumption of egg products provides comparable outcomes in long-term metabolic health. We recognize this RNAseq analysis represents 1 tissue and time point, and other beneficial (or deleterious) effects of WE diet may occur at another time point or in another physiological system. One notable observation is the increased expression of *elovl5* for males fed the HFCON diet compared with all other dietary groups. *elovl5* is a long-chain fatty acid elongase that has been shown previously to respond to dietary changes (31). *elovl5* knockout mice experience onset of fatty liver disease concurrent with a decrease in intercellular arachidonic acid (20:4n−6) and DHA (32). Dietary supplementation of these 2 fatty acids reversed the effects of gene knockout. Increased expression of *elovl5* exclusive to the HFCON-fed male fish and not male fish receiving the WE diet, which contains an equal dietary fat content, suggests a potential impact of egg protein in reducing the negative effects observed in high-fat diets. This hypothesis requires further investigation.

Recent human studies on the impacts of egg consumption on the glycemic response show results consistent with lack of differences observed between egg-containing and non–egg-containing diets (33). The Diabetes and Egg (DIABEGG) Study provided dietary instruction for a 3-mo, low-energy, weight-loss program to diabetic and prediabetic persons. Diet recommendations consisted of either more than 12 eggs/wk or less than 2/wk and estimates of dietary compliance were high. Both dietary recommendation groups provided high protein content with low energy content, but with higher dietary cholesterol in the 12-eggs/wk group. There were no significant differences between the 2 treatments in measures of serum glucose, serum lipids, or markers of inflammation and oxidative stress at study termination or at 12-mo follow up. A study by Baghdasarian et al. (34) obtained participant data from the Framingham Offspring Study and separated them into dietary cholesterol intake amounts. The intake amount of dietary cholesterol was not found to be associated with fasting glucose concentration. This is particularly relevant to our study outcomes given the higher amount of dietary cholesterol contained in whole eggs. In examining liver transcriptome outcomes, we do see differences between our study and others. Zhu et al. (35) formulated EW and WE diets for Golden Syrian hamsters to investigate the impact of liver health from egg-based diets and dietary cholesterol. WE diets, even at the lowest levels of inclusion, increased liver cholesterol, whereas EW diets had no impact. Concomitant to the increased liver cholesterol were expression changes in genes that code for proteins that have direct and indirect interactions with cholesterol—namely, LDL receptor and sterol-regulatory-element-binding protein-2. For our study, liver cholesterol was not measured, but the gene expression changes related to increased cholesterol were detected.

In summation, we recognize the issues associated with human dietary studies, such as limited time span and the confounding variance among individuals. We also recognize that mouse models, while valuable, may have limitations due to differences in lipid processing, including cholesterol. For these reasons, we believe that zebrafish can be a powerful model for the investigation of nutrition-related morbidities, particularly those related to metabolic health, due to the innate similarities of zebrafish to humans in diet and metabolism. Our studies show that replacement of well-established animal protein sources with an egg alternative does not impact growth or metabolic health in zebrafish and, if translated, to human health and metabolism. These data support the broad conclusion that long-term dietary egg consumption is suitable as a replacement of other sources of dietary protein, with potential benefits to be determined.

## Supplementary Material

nzab134_Supplemental_Files

## Data Availability

Data are available from authors upon request.

## Notes

Supported by the Egg Nutrition Center (award proposal number 512304) and the University of Alabama at Birmingham Nutrition Obesity Research Center Lab Animal Nutrition Core (P30DK056336).

Author disclosures: The authors report no conflicts of interest. The project design was submitted to the Egg Nutrition Center for approval. Neither funding group participated in whole or part in data collection, analysis, or interpretation. The final manuscript was submitted without approval of our funding agencies.

Supplemental Figures 1–3 and a Supplemental Data File are available from the “Supplementary data” link in the online posting of the article and from the same link in the online table of contents at https://academic.oup.com/cdn/.

Abbreviations used: BCAA, branched-chain amino acid; CON, fish and casein protein source control diet; DEG, differentially expressed gene; DIO, diet-induced obesity; DPF, days post-fertilization; *elovl5*,elongation of very long chain fatty acids protein 5; EW, egg white; *gpx4a*, glutathione peroxidase 4a; HFCON, high-fat fish and casein protein source diet; PCA, principal component analysis; RNAseq, RNA sequencing; *rpl13a*,ribosomal protein L13a; *ucp2*, uncoupling protein 2; WE, whole egg; WG, wheat gluten.

## References

[1] Larsen TM , Dalskov SM, van Baak M, Jebb SA, Papadaki A, Pfeiffer AF, Martinez JA, Handjieva-Darlenska T, Kunešová M, Pihlsgård M et al. Diets with high or low protein content and glycemic index for weight-loss maintenance. N Engl J Med. 2010;363(22):2102–13. 21105792 10.1056/NEJMoa1007137 PMC3359496

[2] Sargrad KR , Homko C, Mozzoli M, Boden G. Effect of high protein vs high carbohydrate intake on insulin sensitivity, body weight, hemoglobin A1c, and blood pressure in patients with type 2 diabetes mellitus. J Am Diet Assoc. 2005;105(4):573–80. 15800559 10.1016/j.jada.2005.01.009

[3] McAuley KA , Hopkins CM, Smith KJ, McLay RT, Williams SM, Taylor RW, Mann JI. Comparison of high-fat and high-protein diets with a high-carbohydrate diet in insulin-resistant obese women. Diabetologia. 2005;48(1):8–16. 15616799 10.1007/s00125-004-1603-4

[4] Gannon MC , Nuttall FQ. Effect of a high-protein, low-carbohydrate diet on blood glucose control in people with type 2 diabetes. Diabetes. 2004;53(9):2375–82. 15331548 10.2337/diabetes.53.9.2375

[5] Malik VS , Li Y, Tobias DK, Pan A, Hu FB. Dietary protein intake and risk of type 2 diabetes in US men and women. Am J Epidemiol. 2016;183(8):715–28. 27022032 10.1093/aje/kwv268 PMC4832052

[6] Zhang Y , Kobayashi H, Mawatari K, Sato J, Bajotto G, Kitaura Y, Shimomura Y. Effects of branched-chain amino acid supplementation on plasma concentrations of free amino acids, insulin, and energy substrates in young men. J Nutr Sci Vitaminol. 2011;57(1):114–17. 21512300 10.3177/jnsv.57.114

[7] Shah SH , Crosslin DR, Haynes CS, Nelson S, Turer CB, Stevens RD, Muehlbauer MJ, Wenner BR, Bain JR, Laferrere B, Gorroochurn P. Branched-chain amino acid levels are associated with improvement in insulin resistance with weight loss. Diabetologia. 2012;55(2):321–30. 22065088 10.1007/s00125-011-2356-5 PMC3667157

[8] Lopez-Legarrea P , Fuller NR, Martinez JA, Caterson ID, Zulet MA. The influence of Mediterranean, carbohydrate and high protein diets on gut microbiota composition in the treatment of obesity and associated inflammatory state. Asia Pac J Clin Nutr. 2014;23(3):360. 25164445 10.6133/apjcn.2014.23.3.16

[9] Huopalahti R , Anton M, López-Fandiño R, Schade R. Bioactive egg compounds. Berlin, Heidelberg: Springer; 2007.

[10] Baron F , Rehault S. Compounds with antibacterial activity. In: Bioactive egg compounds. Huopalahti R, Anton M, López-Fandiño R, Schade R (editors). Berlin, Heidelberg: Springer; 2007. p. 191–8.

[11] López-Fandiño R , Recio I, Ramos M. Egg-protein-derived peptides with antihypertensive activity. In: Bioactive egg compounds. Huopalahti R, Anton M, López-Fandiño R, Schade R (editors). Berlin, Heidelberg: Springer; 2007. p. 199–211.

[12] Guérin-Dubiard C , Castellani O, Anton M. Egg compounds with antioxidant and mineral binding properties. In: Bioactive egg compounds. Huopalahti R, Anton M, López-Fandiño R, Schade R (editors). Berlin, Heidelberg: Springer; 2007. p. 223–8.

[13] Lieschke GJ , Currie PD. Animal models of human disease: zebrafish swim into view. Nat Rev Genet. 2007;8(5):353. 17440532 10.1038/nrg2091

[14] Fowler LA , Williams MB, D'Abramo LR, Watts SA. Zebrafish nutrition—moving forward. In: The zebrafish in biomedical research. Cartner SC, Eisen JS, Farmer SC, Guillemin KJ, Kent ML, Sanders GE (editors). London, UK: Elsevier; 2020. p. 379–401.

[15] Fowler LA , Williams MB, Dennis-Cornelius LN, Farmer S, Barry RJ, Powell ML, Watts SA. Influence of commercial and laboratory diets on growth, body composition, and reproduction in the zebrafish *Danio rerio*. Zebrafish. 2019;16(6):508–21. 31381491 10.1089/zeb.2019.1742 PMC6916735

[16] Oka T , Nishimura Y, Zang L, Hirano M, Shimada Y, Wang Z, Umemoto N, Kuroyanagi J, Nishimura N, Tanaka T. Diet-induced obesity in zebrafish shares common pathophysiological pathways with mammalian obesity. BMC Physiol. 2010;10(1):1–13. 20961460 10.1186/1472-6793-10-21 PMC2972245

[17] Zang L , Maddison LA, Chen W. Zebrafish as a model for obesity and diabetes. Front Cell Dev Biol. 2018;6:91. 30177968 10.3389/fcell.2018.00091 PMC6110173

[18] Folch J , Lees M, Stanley GS. A simple method for the isolation and purification of total lipides from animal tissues. J Biol Chem. 1957;226(1):497–509. 13428781

[19] Eames SC , Philipson LH, Prince VE, Kinkel MD. Blood sugar measurement in zebrafish reveals dynamics of glucose homeostasis. Zebrafish. 2010;7(2):205–13. 20515318 10.1089/zeb.2009.0640 PMC2882991

[20] Andrews S . FastQC: a quality control tool for high throughput sequence data. Cambridge (UK): Babraham Bioinformatics, Babraham Institute; 2010.

[21] Bolger AM , Lohse M, Usadel B. Trimmomatic: a flexible trimmer for Illumina sequence data. Bioinformatics. 2014;30(15):2114–20. 24695404 10.1093/bioinformatics/btu170 PMC4103590

[22] Dobin A , Davis CA, Schlesinger F, Drenkow J, Zaleski C, Jha S, Batut P, Chaisson M, Gingeras TR. STAR: ultrafast universal RNA-seq aligner. Bioinformatics. 2013;29(1):15–21. 23104886 10.1093/bioinformatics/bts635 PMC3530905

[23] Love MI , Huber W, Anders S. Moderated estimation of fold change and dispersion for RNA-seq data with DESeq2. Genome Biol. 2014;15(12):1–21. 10.1186/s13059-014-0550-8 PMC4302049 25516281

[24] Harris ML , Fufa TD, Palmer JW, Joshi SS, Larson DM, Incao A, Gildea DE, Trivedi NS, Lee AN, Day CP et al. A direct link between MITF, innate immunity, and hair graying. PLoS Biol. 2018;16(5):e2003648. 29723194 10.1371/journal.pbio.2003648 PMC5933715

[25] Liao Y , Smyth GK, Shi W. featureCounts: an efficient general purpose program for assigning sequence reads to genomic features. Bioinformatics. 2014;30(7):923–30. 24227677 10.1093/bioinformatics/btt656

[26] Marini F , Binder H. pcaExplorer: an R/Bioconductor package for interacting with RNA-seq principal components. BMC Bioinformatics. 2019;20(1):1–8. 31195976 10.1186/s12859-019-2879-1 PMC6567655

[27] Elli L , Branchi F, Tomba C, Villalta D, Norsa L, Ferretti F, Roncoroni L, Bardella MT. Diagnosis of gluten related disorders: celiac disease, wheat allergy and non-celiac gluten sensitivity. World J Gastroenterol. 2015;21(23):7110. 26109797 10.3748/wjg.v21.i23.7110 PMC4476872

[28] Bhowmick SK , Levens KL, Rettig KR. Hyperosmolar hyperglycemic crisis: an acute life-threatening event in children and adolescents with type 2 diabetes mellitus. Endocr Pract. 2005;11(1):23–9. 16033732 10.4158/EP.11.1.23

[29] Yeannes MI , Almandos ME. Estimation of fish proximate composition starting from water content. J Food Compos Anal. 2003;16(1):81–92.

[30] Jobling M . National Research Council (NRC): Nutrient requirements of fish and shrimp. Washington (DC): The National Academies Press; 2011.

[31] Tripathy S , Jump DB. Elovl5 regulates the mTORC2-Akt-FOXO1 pathway by controlling hepatic cis-vaccenic acid synthesis in diet-induced obese mice. J Lipid Res. 2013;54(1):71–84. 23099444 10.1194/jlr.M028787 PMC3520542

[32] Moon Y-A , Hammer RE, Horton JD. Deletion of ELOVL5 leads to fatty liver through activation of SREBP-1c in mice. J Lipid Res. 2009;50(3):412–23. 18838740 10.1194/jlr.M800383-JLR200 PMC2638104

[33] Fuller NR , Sainsbury A, Caterson ID, Denyer G, Fong M, Gerofi J, Leung C, Lau NS, Williams KH, Januszewski AS et al. Effect of a high-egg diet on cardiometabolic risk factors in people with type 2 diabetes: the Diabetes and Egg (DIABEGG) Study—randomized weight-loss and follow-up phase. Am J Clin Nutr. 2018;107(6):921–31. 29741558 10.1093/ajcn/nqy048

[34] Baghdasarian S , Lin HP, Pickering RT, Mott MM, Singer MR, Bradlee ML, Moore LL. Dietary cholesterol intake is not associated with risk of type 2 diabetes in the Framingham Offspring Study. Nutrients. 2018;10(6):665. 10.3390/nu10060665 PMC6024792 29794966

[35] Zhu H , He Z, Kwek E, Liu J, Hao W, Liang N, Zhao Y, Ma KY, He WS, Chen ZY. Dose-dependent increases in liver cholesterol but not plasma cholesterol from consumption of one to five whole eggs and no effects from egg whites on liver or plasma cholesterol in hamsters. J Agric Food Chem. 2018;66(48):12805–14. 30415537 10.1021/acs.jafc.8b04730

