# Supplementary material for: Effect of Long-Term Consumption of Poultry Egg Products on Growth, Body Composition, and Liver Gene Expression in Zebrafish, Danio rerio
Source: Curr Dev Nutr. 2021 Dec 24;5(12):nzab134. doi: 10.1093/cdn/nzab134 (PMC8718328; doi:10.1093/cdn/nzab134)
Supplement: nzab134_Supplemental_Files [file nzab134_supplemental_files.zip › Supplemental_Figures.docx]

EFFECT OF LONG-TERM CONSUMPTION OF POULTRY EGG PRODUCTS ON GROWTH, BODY COMPOSITION AND LIVER GENE EXPRESSION IN ZEBRA FISH, Danio rerio. Williams et al., Online Supplementary Material


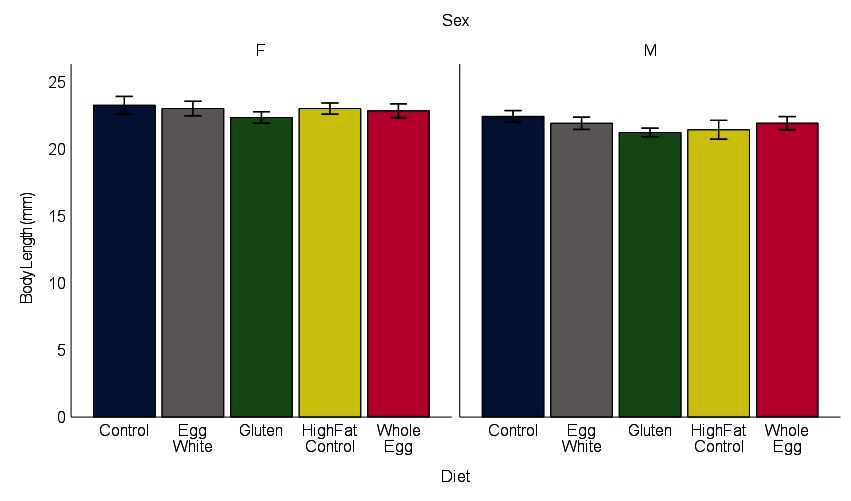
Supplemental Figure 1. Bars represent mean and standard error of total body moisture (percent) for male and female zebrafish measured at the end of 32 weeks on the assigned diets (n=8 tanks, 10 fish per tank for each diet treatment). There were no statistically significant differences among treatments.


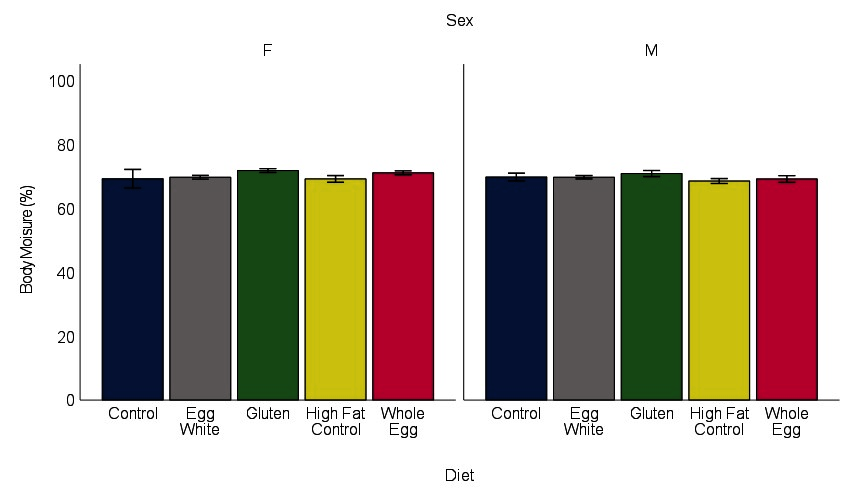


Supplemental Figure 2. Bars represent mean and standard error of standard body length (mm) for male and female zebrafish measured at the end of 32 weeks on the assigned diets (n=8 tanks, 10 fish per tank for each diet treatment). There were no statistically significant differences among treatments.


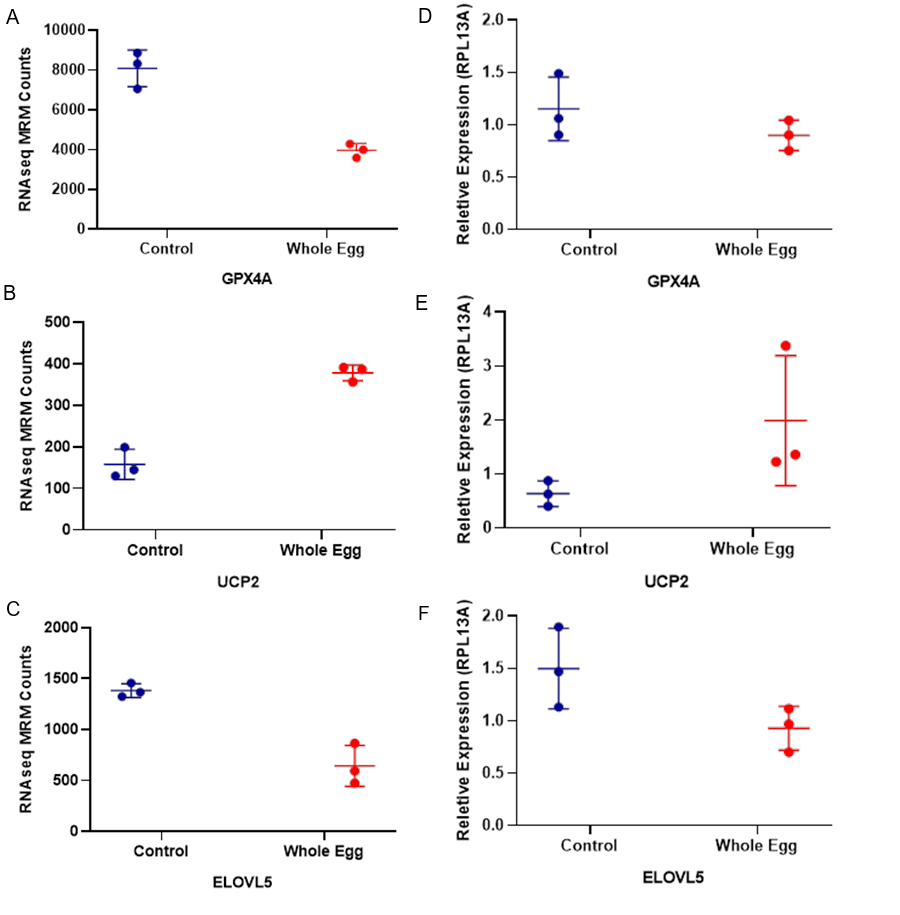


Supplemental Figure 3. RNAseq counts (A, B, and C) and qPCR expression (D, E, and F) of Control and Whole Egg samples (n=3) excluding samples Control-4 and Whole Egg-1 which were overlapping.
